# Evaluation of surface roughness of enamel after various bonding and clean-up procedures on enamel bonded with three different bonding agents: An *in-vitro* study

**DOI:** 10.4317/jced.53237

**Published:** 2017-05-01

**Authors:** Amit Goel, Atul Singh, Tarun Gupta, Ramandeep-Singh Gambhir

**Affiliations:** 1 Post Graduate Student, Dept. of Orthodontics, KD Dental College and Hospital, Mathura, UP; 2 Reader, Dept. of Orthodontics, KD Dental College and Hospital, Mathura, UP; 3 Sr. Lecturer, Dept. of Public Health Dentistry, MM University, Mullana, Ambala; 4 Reader and Head, Dept. of Public Health Dentistry, Rayat and Bahra Dental College and Hospital, Mohali

## Abstract

**Background:**

The purpose of this study was to analyze and compare the enamel surface roughness before bonding and after debonding, to find correlation between the adhesive remnant index and its effect on enamel surface roughness and to evaluate which clean-up method is most efficient to provide a smoother enamel surface.

**Material and Methods:**

135 premolars were divided into 3 groups containing 45 premolars in each group. Group I was bonded by using moisture insensitive primer, Group II by using conventional orthodontic adhesive and Group III by using self-etching primer. Each group was divided into 3 sub-groups on the basis of type of clean-up method applied i,e scaling followed by polishing, tungsten carbide bur and Sof-Lex disc. Enamel surface roughness was measured and compared before bonding and after clean-up.

**Results:**

Evaluation of pre bonding and post clean-up enamel surface roughness (Ra value) with the t test showed that Post clean-up Ra values were greater than Pre bonding Ra values in all the groups except in teeth bonded with self-etching primer cleaned with Sof-Lex disc. Reliability of ARI score taken at different time interval tested with Kruskal Wallis test suggested that all the readings were reliable.

**Conclusions:**

No clean-up procedure was able to restore the enamel to its original smoothness. Self-etching primer and Sof-Lex disc clean-up method combination restored the enamel surface roughness (Ra value) closest to its pre-treatment value.

** Key words:**Enamel surface roughness, clean-up method, adhesive remnant index.

## Introduction

At completion of ﬁxed appliance therapy, one of the orthodontist’s primary concerns is to return the enamel surface to its original state as far as possible. The ideal would be minimal enamel loss at each stage of the bonding, debonding, and enamel clean-up process and the production of an enamel surface with the same degree of roughness or smoothness as the original, untreated tooth (1). During bracket removal, bond failure can occur at the adhesive-enamel interface or at the adhesive-bracket interface (adhesive failure), or within the adhesive (cohesive failure). Generally, bracket failure is a combination of adhesive and cohesive failures, the latter resulting in retention of material on the enamel and bracket surfaces (mixed failure) (2).

A certain amount of enamel loss is almost inevitable because of the failure of micromechanical bond between the composite resin bonding agent and the acid-etched enamel (3-5). At present, no universally approved protocol has been established for the removal of adhesive resin after debracketing, and no instrument can achieve complete composite removal without affecting the enamel surface (6,7).

The exploration of an efficient and safe method of adhesive resin removal following debonding has attracted the interest of many investigators, resulting in the introduction of a wide range of instruments and procedures. Residual adhesive on the enamel surface after debonding can be removed in various ways, including hand instruments (pliers and scalers), various burs, Sof-lex discs, ultrasonic devices, air abrasion units and lasers but studies have shown that some recommended modalities damage enamel surfaces (6,7). It has been reported that significant amount of enamel loss and irreversible damage to enamel can occur due to mechanical removal of remaining composite after debonding procedure (8,9).

Assessment of the effectiveness of rotary instruments was limited to inspecting the surface under scanning electron microscopy (SEM) to see the morphology of the enamel surface (7). However, such investigations are subjective and cannot be used alone to judge the reliability of a clean-up protocol. Alternative techniques should be used, such as proﬁlometry (surface roughness parameters). The roughness parameters measured via profilometry include: Average roughness, Root mean square roughness, Maximum roughness depth and Mean roughness depth (7). Proﬁlometry provides quantitative results, whereas SEM affords a more subjective inspection (10,11).

The hypothesis tested in this study is that the method of clean up may affect enamel roughness. Therefore, the purpose of this study was to analyze enamel surface roughness before bonding with various adhesives and after debonding followed by various clean-up methods, and to provide a quantitative data on which clean-up method is most efficient to provide a smoother enamel surface and to correlate between the adhesive remnant after debonding and its effect on surface roughness.

## Material and Methods

-Study sample 

**Figure 1:**
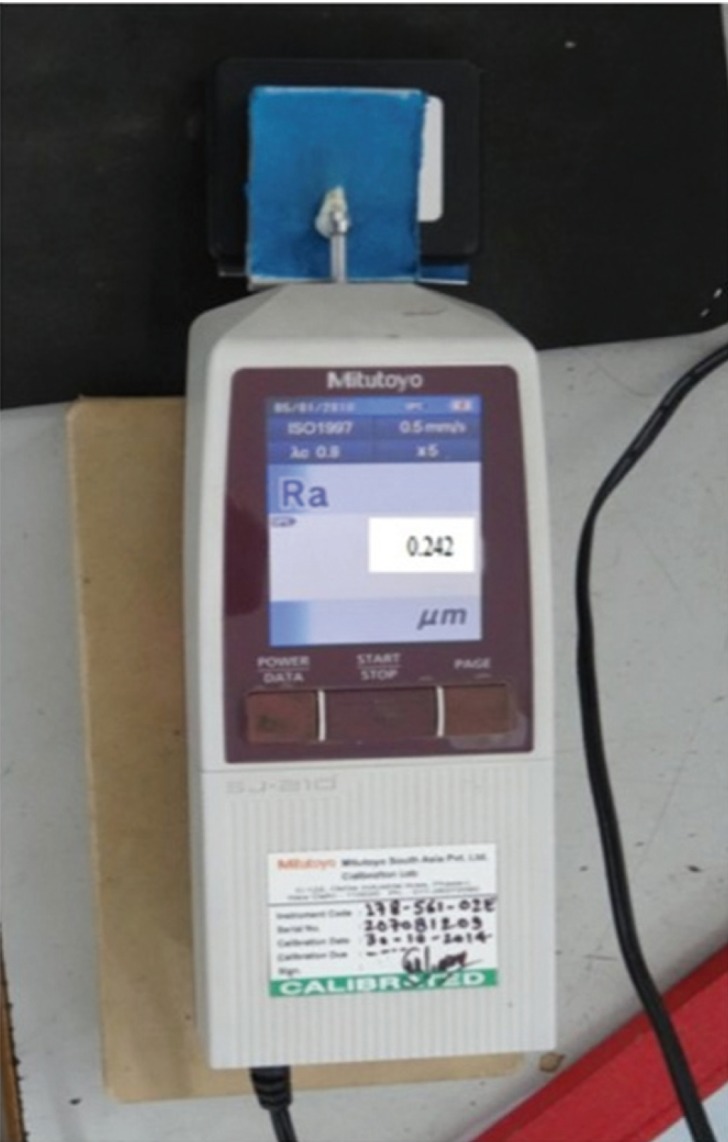
Extracted premolars mounted in coloured acrylic custom made blocks.

Before the start of the study ethical approvals were sought from the institutional review board for conducting the study. A total of 135 extracted premolars were selected, cleaned and stored in distilled water at room temperature. The inclusion criteria included: teeth extracted within 45 days, teeth with no caries or restorations on the buccal surface, teeth having no enamel defects, hypocalcifications or fluorosis on the buccal surface and teeth with no visible cracks on the buccal surface. The extracted premolars were mounted in acrylic custom made blocks which were colour coded and were divided into three different groups on the basis of the type of adhesive used (Fig. 1).

-Group Division 

Group 1 = Blue (Moisture insensitive primer) (Transbond MIP, 3M Unitek)

Group 2 = Yellow (Conventional orthodontic adhesive) (Transbond XT, 3M Unitek)

Group 3 = Red (Self etching primer) (Transbond SEP, 3M Unitek)

-Estimation of surface roughness

**Figure 2:**
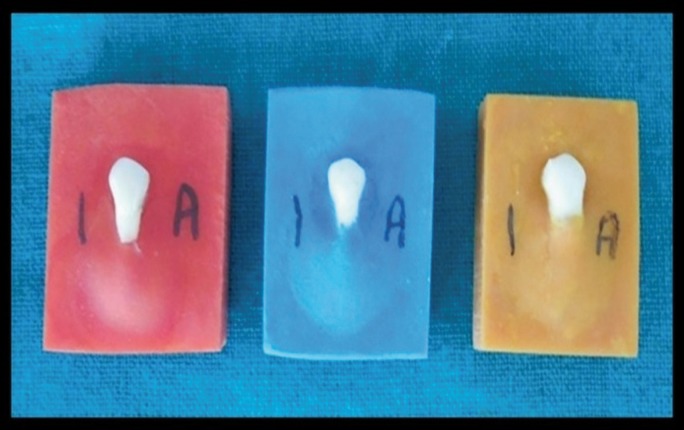
Mitutoyo Surface Roughness Tester.

After mounting, the buccal enamel surfaces were pumiced, washed thoroughly and dried with moisture free spray to remove the organic layer. On the buccal surface of all the teeth two layers of nail varnish were applied keeping a circular area of 3 mm in diameter on the middle part exposed to provide the same area of measurement in different treatment stages and to avoid contamination of the prospective bonding buccal surface. Before bonding the labial enamel surface of all the samples were subjected to Mitutoyo Surface Roughness Tester (SJ-210) (Fig. 2) for evaluation of surface roughness, which was indicated by Ra value for each particular tooth. Ra value is a commonly used parameter for characterizing surface roughness of a tooth. It describes the overall surface roughness, and the unit for measuring the Ra value is µm.

-Application of adhesive materials 

All the selected teeth were cleaned with tap water prior to bonding. Groups 1 and 2 were subjected to acid etching with 37% orthophosphric acid gel (Ultradent Product, Inc., South Jordan, UT, USA) for 30 seconds, rinsed and dried. A chalky appearance of the enamel surface verified successful etching. Then group 1 was bonded using moisture insensitive primer, group 2 by using conventional orthodontic adhesive and group 3 by using self-etching primer using a micro-brush and lightly dried with compressed air. In all the groups, the primer was cured for 10 seconds using mini LED black (Satelec Aceteon) curing light. Stainless steel Pre Adjusted Edgewise premolar brackets (Gemini Twin brackets, 3M Unitek) with slot size of 0.022” x 0.028” were bonded on each specimen using light cure adhesive paste (Transbond XT, 3M Unitek). The brackets were placed in the middle of the tooth in occlusal-gingival and mesial-distal directions, and seated with firm pressure. Excess adhesive resin was carefully removed with an explorer. The curing time was 40 seconds from occlusal, gingival, mesial and distal directions (10 seconds each) as per the manufacturer’s instruction.

-Debonding and evaluation of remaining adhesive 

All the cured specimens were kept for 24 hrs before debonding. Debonding was carried out by debonding plier by applying a gentle squeezing force on the outer wings of the bracket. After debonding, Adhesive Remnant Index (ARI) (12) was evaluated by visual observation of the specimen with naked eye at different time intervals so as to minimize the error. The ARI score was taken at the time of debonding, after 24 hrs of debonding and after 48 hrs of debonding.

Score 0,- 0% of adhesive remaining on the tooth;

Score 1, - ≤ 50% of adhesive remaining on the tooth;

Score 2, - > 50% of adhesive remaining on the tooth;

Score 3, - 100% of adhesive remaining on the tooth.

Each group was further subdivided into 3 sub groups on the basis of the type of clean-up method applied for the removal of remaining adhesive resin left after debonding, which are as follow.

Group A: Ultra sonic piezo scaling followed by pumice application 

Group B: 12 fluted Tungsten carbide bur

Group C: Sof-Lex disc (3M ESPE)

The Sof-Lex discs (3M ESPE) were used in decreasing order of coarseness starting initially with coarse disc followed by medium, fine and finally super fine disc. Final polishing was carried out using rubber cup and zirconium silicate paste (Astek Innovations Ltd, Altrincham, United Kingdom). After complete removal of the remaining composite resin from the enamel surface of all the teeth, which was veriﬁed by visual inspection of the teeth under a dental operating light under wet and dry condition, all the samples were once again subjected to Mitutoyo Surface Roughness Tester (SJ-210) for evaluation of surface roughness for each particular tooth.

To assess the quality of the enamel surface after the debonding procedures and to find any correlation between the ARI score and post clean-up enamel surface roughness (Ra value), the sub-groups under each group were combined and treated as one group.

-Statistical Analysis 

The data obtained was subjected to statistical analysis which was performed using SPSS (Statistical Package for Social Science) version 16.0 for Windows. One-way Analysis of Variance (ANOVA), Kruskal Wallis test, Post hoc Tukey test Honestly Significant Difference (HSD) tests, t-test and Pearson‘s correlation test were used for statistical analysis to compare the enamel surface roughness of teeth before bonding and after debonding and clean-up with various adhesives, which clean-up method produced a smoother enamel, reliability of the ARI score and to correlate between the ARI & post clean-up enamel surface roughness. The level of statistical significance was set at 95% (*p*=0.05).

## Results

Post clean-up enamel surface roughness (Ra value) was greater than Pre bonding enamel surface roughness (Ra value) in all the groups. This difference in Ra was found to be highly statistically significant (*p*=0.000) except in teeth bonded with self-etching primer cleaned with Sof-Lex disc (*p*=0.098) ( Table 1). Enamel surface roughness was found to be different when same adhesive was removed with different clean-up methods and this difference in enamel surface roughness (Ra value) was found to be statistically significant (*p*=0.000) ( Table 2). Enamel surface roughness (Ra value) was different for three different adhesives after clean-up but this difference was statistically significant only in the group cleaned with Sof-Lex disc (Sub-group C, *p*=0.030). This difference in other clean-up methods was inconclusive (Sub-groups A&B).

**Table 1:**
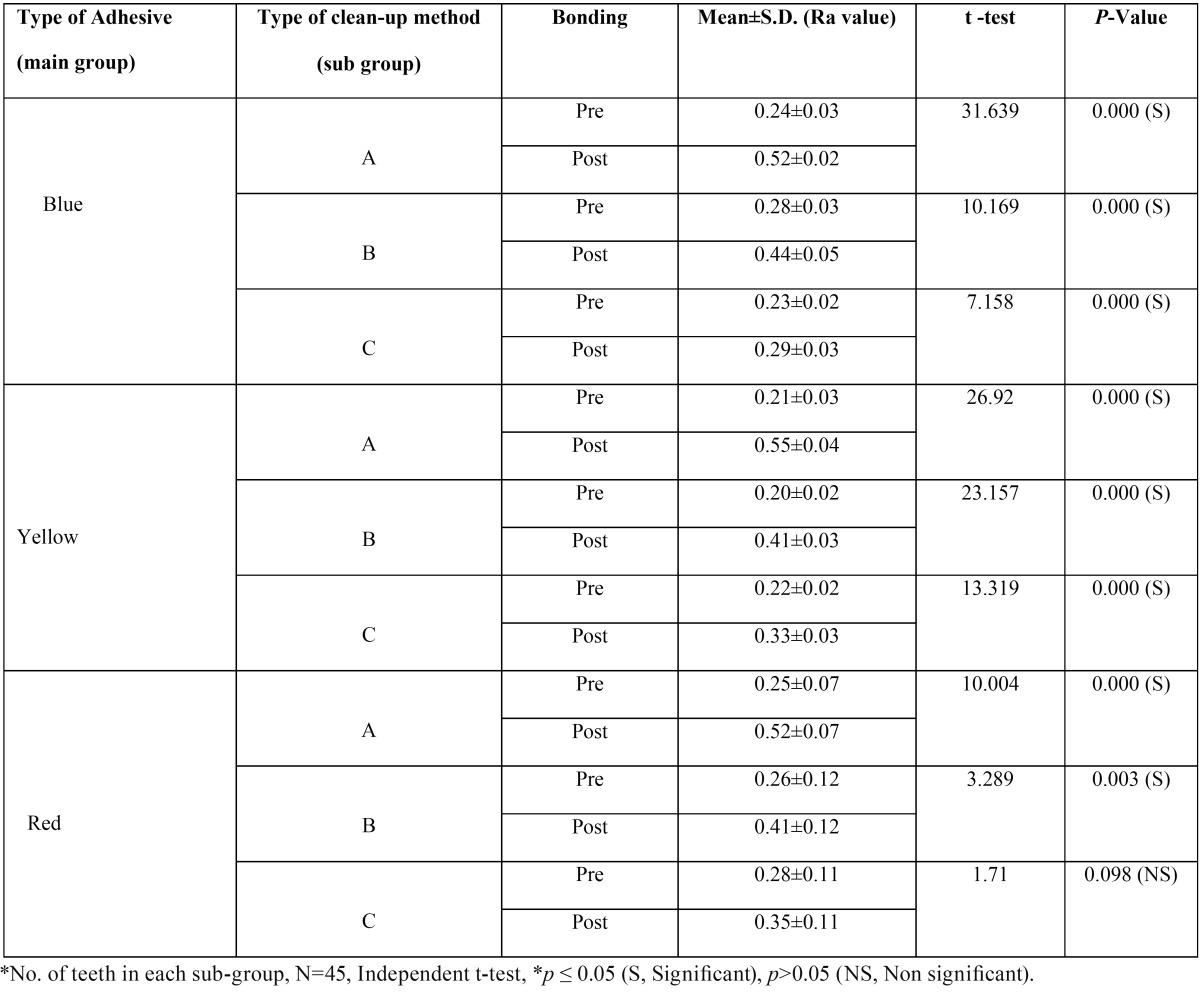
Comparison of enamel surface roughness (Ra value) of individual adhesives pre bonding and after 3 different types of clean-up procedures.

**Table 2:**
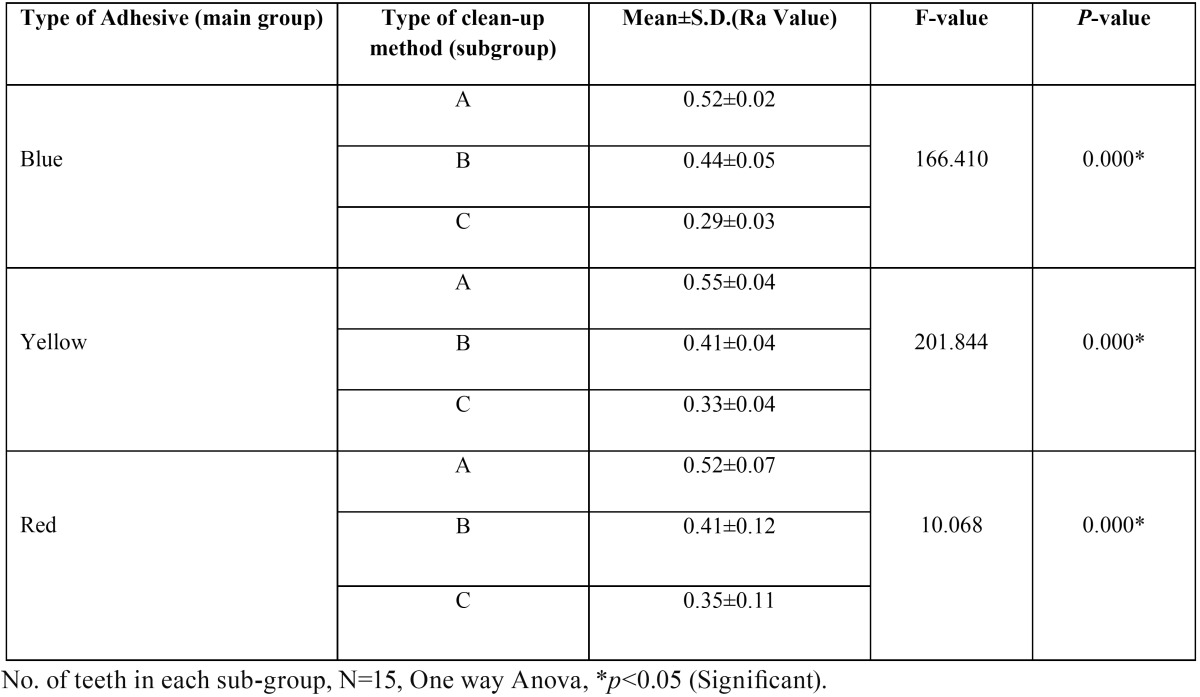
Shows analysis of variance of enamel surface roughness (Ra value) post clean-up of individual adhesives when subjected to 3 different types of clean-up methods.

 Table 3 and  Table 4 show multiple comparisons between three individual clean-up methods and between three individual adhesives respectively. It reveals that self-etching primer and Sof-Lex disc clean-up method combination restored the enamel surface roughness (Ra value) closer to its pre-treatment value. Second best combination was that of the self-etching primer and tungsten carbide bur.

**Table 3:**
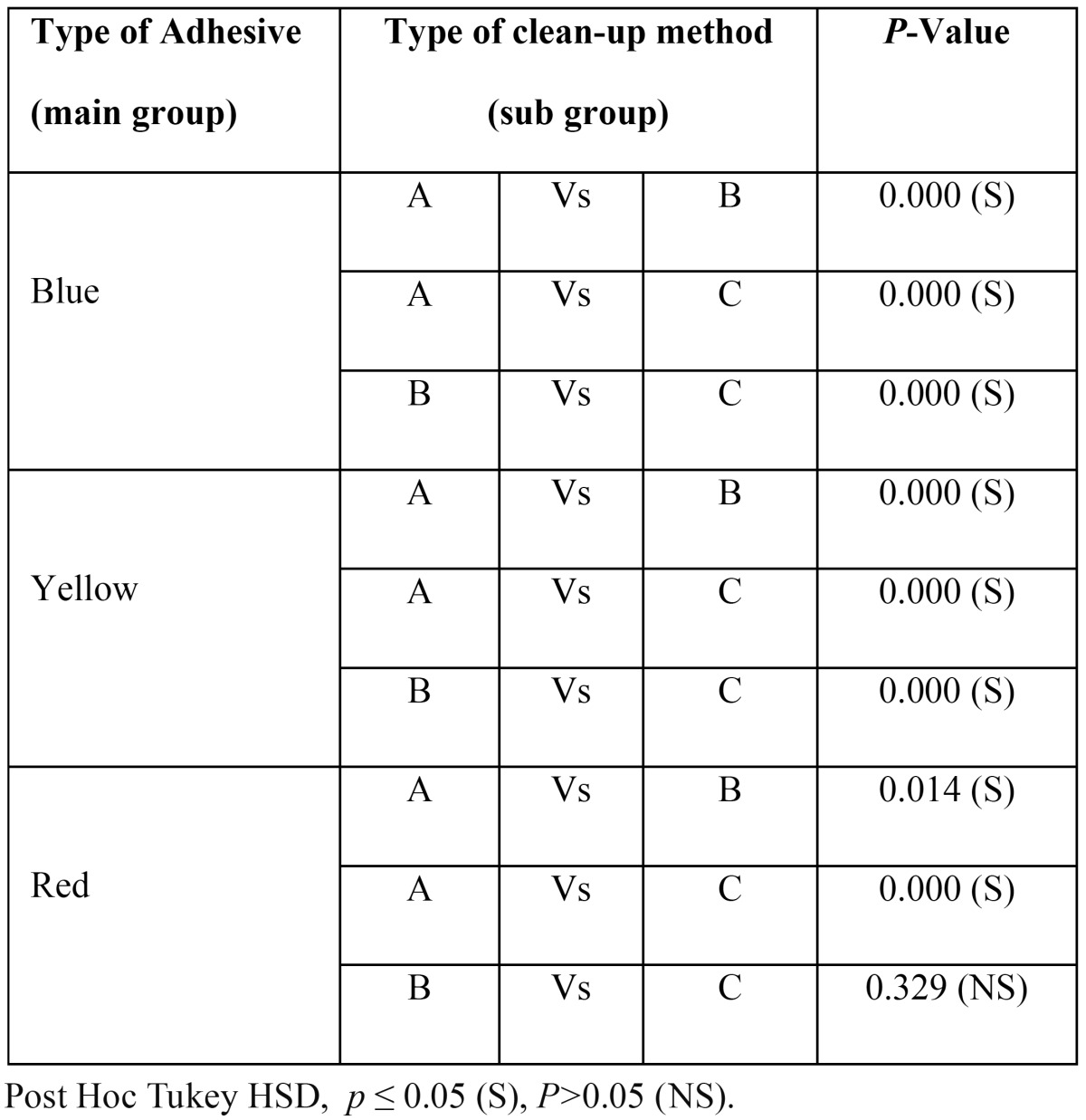
Shows multiple comparisons of enamel surface roughness (Ra value) between 3 different types of clean-up methods subjected on individual adhesives system.

**Table 4:**
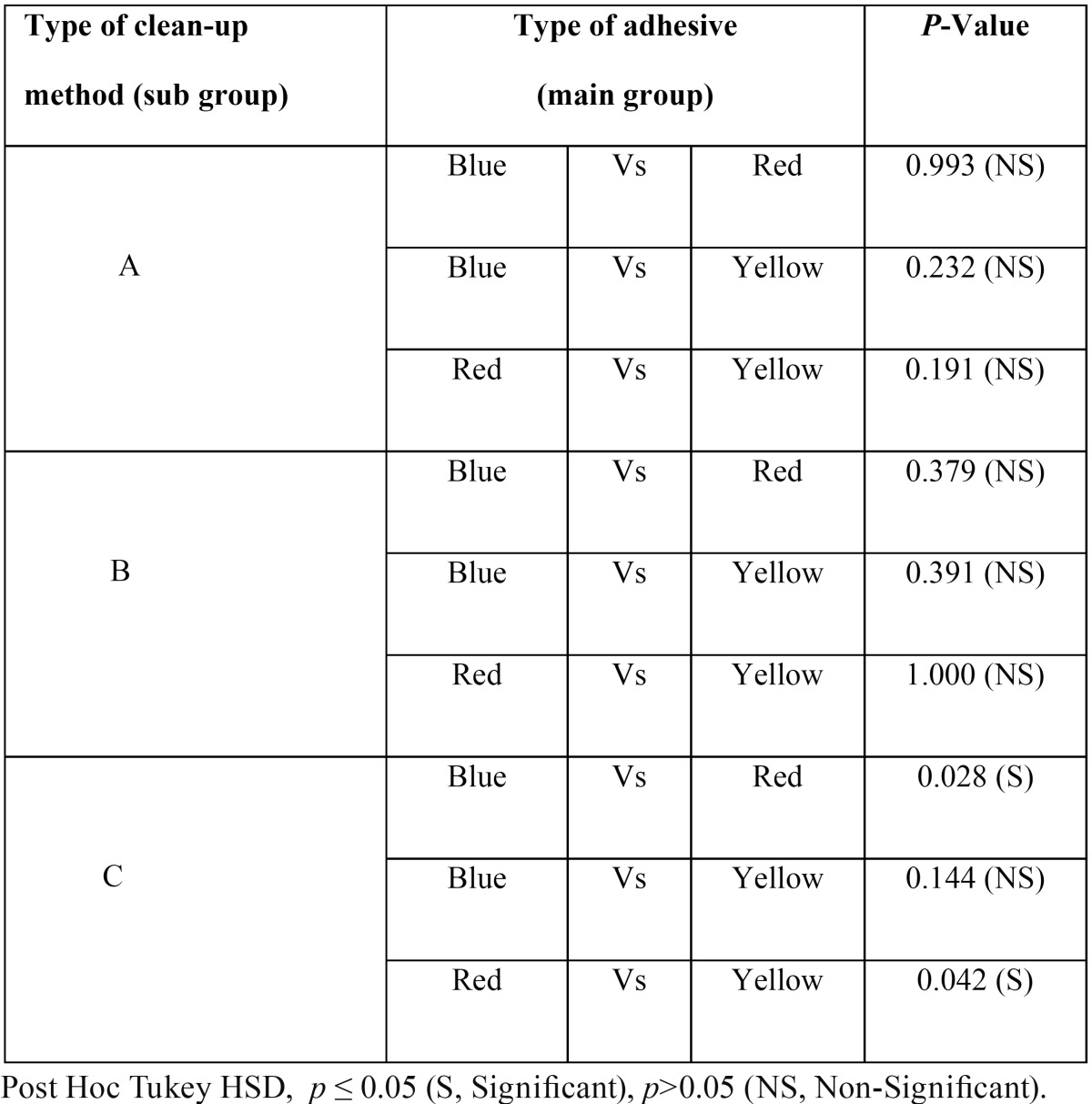
Shows multiple comparisons of enamel surface roughness (Ra value) between the 3 different types of adhesives subjected with individual clean-up methods.

 Table 5 shows the reliability of ARI score taken on different time interval tested with Kruskal Wallis test and the results suggest that there was no significant difference between the different readings of ARI score. Hence, all the readings were considered reliable.

**Table 5:**
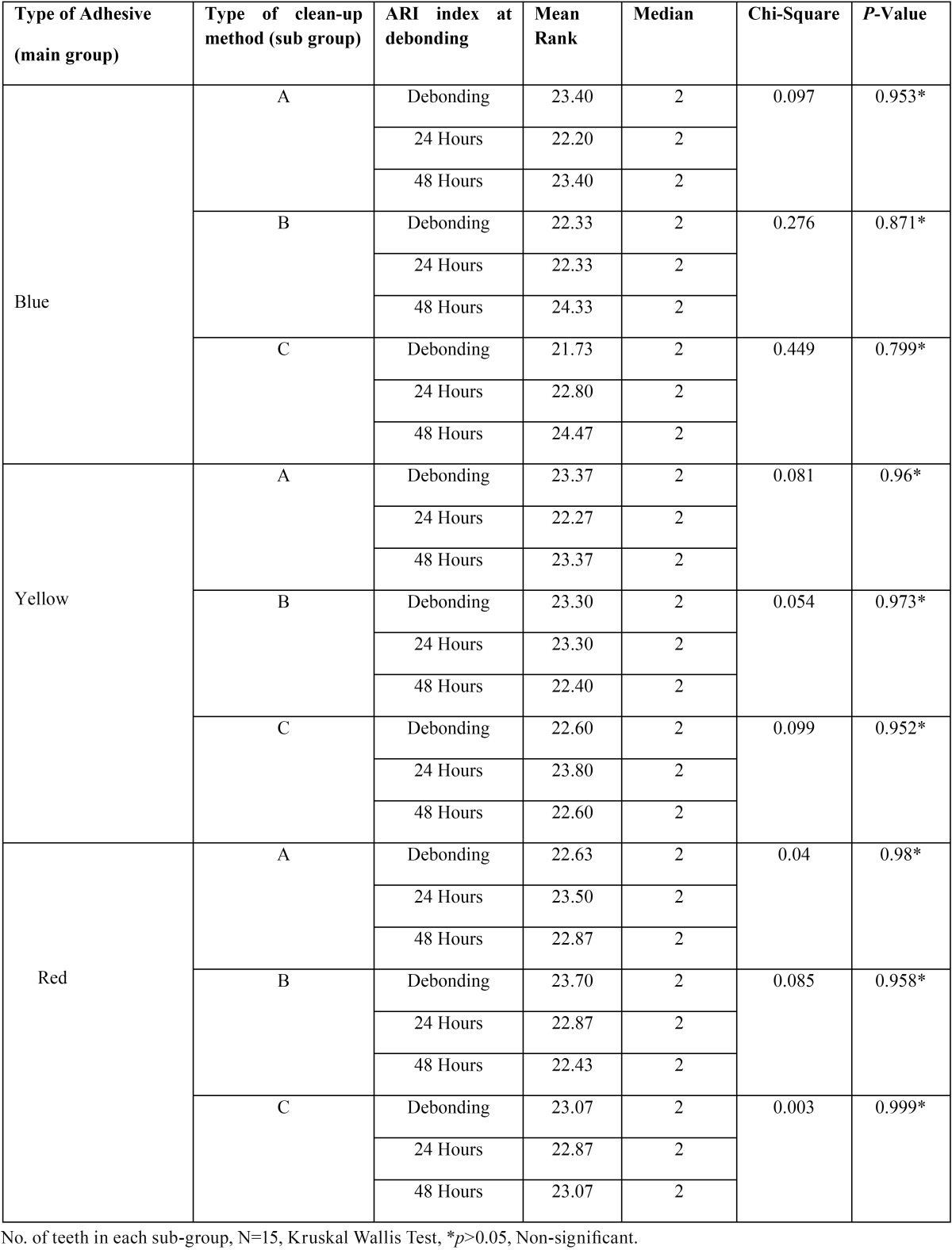
Shows the reliability of ARI score taken on different time intervals when bonded with 3 different types of adhesives.

 Table 6 shows correlation between the ARI score and post clean-up enamel surface roughness (Ra value) compared by Pearson’s correlation test. ARI score showed positive correlation with the post clean-up enamel surface roughness (Ra value) of teeth when bonded with self-etching primer and subjected to any types of clean-up method but still it was statistically non significant (*p*>0.05) whereas teeth bonded with moisture insensitive primer or conventional orthodontic adhesive and cleaned by any type of clean-up method showed negative correlation which was also statistically non-significant (*p*>0.05).

**Table 6:**
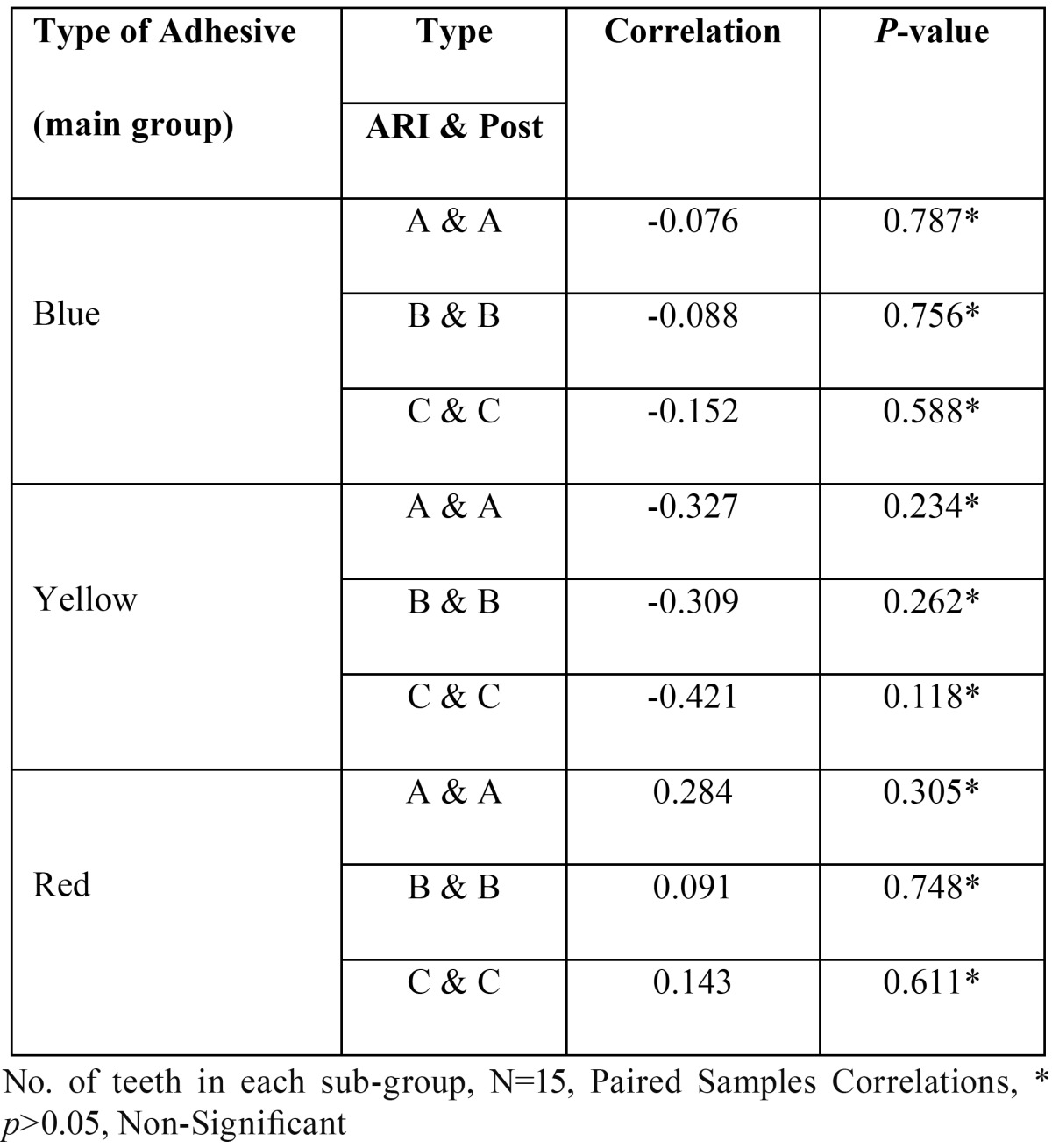
Shows correlation between the ARI score and post clean-up enamel surface roughness (Ra value) of individual adhesives when subjected to 3 different types of clean-up methods.

## Discussion

Clinical orthodontic treatment has been revolutionized during the past decades with the advent of direct bonding. Placement of orthodontic attachments on the surface of teeth can be accomplished by using bonding materials. However, unlike when used for restorative dentistry, these materials must be removed from the surface of enamel at the completion of orthodontic treatment. The concern over debonding-induced enamel surface alterations derives from the importance of the uppermost layer of enamel attributed to its hardness, higher mineral content and more fluoride relative to deeper zones (7).

Extracted human teeth are very difficult to collect, store, and standardize. The teeth used in this study were premolar teeth previously extracted for orthodontic purposes. Since the enamel surface was the focus of this investigation, great care was taken to select the surfaces with the least amount of pre-existing damage and defect which was the basis for the inclusion criteria.

Debonding and cleanup are operator-dependent procedures, so the results might vary between operators. In order to minimize this error, only one operator carried out all the clinical procedures in the present study. Results of the present study revealed that post clean-up enamel surface roughness (Ra value) was more than the pre bonding enamel surface roughness (Ra value) in all the groups. This means that no clean-up method was able to fully restore the enamel surface roughness to its original state.

Only in case of self-etching primer cleaned with Sof-Lex disc group, the difference in pre bonding and post clean-up enamel surface roughness (Ra value) was non-significant i,e. Sof-Lex disc was able to restore the enamel surface roughness close to its original state. This finding is similar to the finding of some other study conducted by Ozer *et al.* (6). This may be because Sof-lex discs are used by planar motion where the axis of rotation of the abrasive is perpendicular to the surfaces being smoothed. This type of motion produces smoother surfaces than rotary motion because, discs tend to sand the surfaces without gouging into the material (13). On the other hand, diamond and carbide burs grind into the surface because they are used by rotary motion where the axis of rotation is parallel to the surfaces being smoothed (14). However, contrasting results were obtained in a study where Sof-Lex disc followed by pumice slurry resulted in the roughest enamel surface (15).

Clean-up method efficiency was also checked in our study and we found that scaling was least efficient followed by tungsten carbide bur and then Sof-Lex disc. This finding was evaluated by keeping the other factors constant eg. type of adhesive, types of brackets, etc. Our finding was in contrast with the findings of Krell *et al.* (16) who reported that ultrasonic scaler produces less enamel loss than the high speed tungsten carbide bur. On the contrary, reports of some studies revealed that scaling produces irreversible damage to the enamel surface and greater enamel loss was seen with the ultrasonic scaler than the tungsten carbide bur (1,17). However, findings of another study revealed that the specimens that were cleaned with tungsten carbide bur showed no significant difference in surface irregularity between the different treatment stages (*p*>0.05). Surface roughness increased significantly after clean-up with the diamond bur and the Er:YAG laser (*p*<0.01) (9).

It was reported by some authors that Sof-Lex disc was able to restore the enamel to its original smoothness as compared to tungsten carbide bur used alone or followed with polishing (6). Inter group comparison in the present study also revealed that combination of self-etching primer and Sof-Lex disc clean-up method restored the enamel surface roughness (Ra value) closer to its pre-treatment value. Second best combination was that of the self-etching primer and tungsten carbide bur. This is because tungsten carbide bur left deep scares on the enamel during clean-up. This finding is analogous to our study. Zarrinia *et al.* (18) stated that carbide burs at high speed proved to be efficient in residual resin removal but failed to produce a satisfactory enamel surface. Moreover using carbide bur is time-consuming and inefficient, and can damage tooth enamel (19). After the removal of residual resin, graded medium, fine, and superfine Sof-Lex finishing disks produced surfaces that could be readily restored satisfactorily after receiving a final polish with a rubber cup and zircate paste (zirconium silicate paste). Scaling gave the same result when used in combination with any adhesives and is found to be the most inefficient method of restoring the enamel surface to its original state.

Teeth bonded with moisture insensitive primer and conventional orthodontic adhesive showed negative correlation with the post clean-up enamel surface roughness. It means that the more the residual adhesive remains on the teeth, less is the post clean-up enamel surface roughness. Only Self etching primer showed positive correlation with the post clean-up enamel surface roughness (Ra value), although the result was inconclusive for all the groups. This may be because the bond failure occurred at the bracket adhesive interface (adhesive failure) or between the adhesive itself (cohesive failure) (2). These have less damaging effect on enamel than the failure occurring at enamel adhesive interface (adhesive failure). Further studies are required to investigate the cause and find the applicable correlation and proper reasoning behind it.

## Conclusions

It can be concluded from the study that-

a) No clean-up procedure was able to restore the enamel to its original smoothness.

b) Sof-Lex disc was the only clean-up method to be able to restore the enamel surface closest to its original state.

c) Self-etching primer and Sof-Lex disc clean-up method combination restored the enamel surface closest to its pre-treatment value.

d) Correlation between ARI score and post clean-up enamel surface roughness (Ra value) shows non-significant result.
